# Novel Method for Automated Analysis of Retinal Images: Results in Subjects with Hypertensive Retinopathy and CADASIL

**DOI:** 10.1155/2015/752957

**Published:** 2015-06-08

**Authors:** Michele Cavallari, Claudio Stamile, Renato Umeton, Francesco Calimeri, Francesco Orzi

**Affiliations:** ^1^Center for Neurological Imaging, Department of Radiology, Brigham and Women's Hospital, Harvard Medical School, 221 Longwood Avenue, Boston, MA 02115, USA; ^2^Department of Neurosciences, Mental Health and Sensory Organs (NESMOS), University of Rome “La Sapienza”, Sant'Andrea Hospital, Via di Grottarossa 1035, 00189 Rome, Italy; ^3^Department of Mathematics and Computer Science (DeMaCS), University of Calabria, 87036 Rende, Italy

## Abstract

Morphological analysis of the retinal vessels by fundoscopy provides noninvasive means for detecting and staging systemic microvascular damage. However, full exploitation of fundoscopy in clinical settings is limited by paucity of quantitative, objective information obtainable through the observer-driven evaluations currently employed in routine practice. Here, we report on the development of a semiautomated, computer-based method to assess retinal vessel morphology. The method allows simultaneous and operator-independent quantitative assessment of arteriole-to-venule ratio, tortuosity index, and mean fractal dimension. The method was implemented in two conditions known for being associated with retinal vessel changes: hypertensive retinopathy and Cerebral Autosomal Dominant Arteriopathy with Subcortical Infarcts and Leukoencephalopathy (CADASIL). The results showed that our approach is effective in detecting and quantifying the retinal vessel abnormalities. Arteriole-to-venule ratio, tortuosity index, and mean fractal dimension were altered in the subjects with hypertensive retinopathy or CADASIL with respect to age- and gender-matched controls. The interrater reliability was excellent for all the three indices (intraclass correlation coefficient ≥ 85%). The method represents simple and highly reproducible means for discriminating pathological conditions characterized by morphological changes of retinal vessels. The advantages of our method include simultaneous and operator-independent assessment of different parameters and improved reliability of the measurements.

## 1. Introduction

It has been known for long time that retinal changes reflect systemic microvascular damage associated with a number of pathological conditions, such as hypertension or diabetes [1]. Because of anatomical and developmental similarities with the central nervous system [2], the retinal vessels are thought to especially mirror the brain microvasculature. In fact, there is evidence that retinal vessel abnormalities are associated with increased risk of stroke [3], stroke mortality [4], cerebral white matter damage [5], carotid atherosclerosis [6], intracranial large artery disease [7], cerebral amyloid angiopathy [8], or Cerebral Autosomal Dominant Arteriopathy with Subcortical Infarcts and Leukoencephalopathy (CADASIL) [9]. Retinal vessels can be easily inspected by fundoscopy, and all these findings together support the use of fundoscopy as a tool for staging or early diagnosis of cerebral small-vessel diseases. However, full exploitation of fundoscopy in clinical settings is limited because quantitative information can hardly be obtained through the observer-driven evaluations currently employed in routine clinical practice.

The recent development of digital imaging techniques allows for impressive capabilities of storage, transfer, and quantitation of retinal images. In the last decade, several computer-assisted methods have been developed to automate the analysis of retinal images [10–15]. Morphometric parameters of the retinal vasculature, such as vessel diameter, tortuosity, and mean fractal dimension (mean-D), constitute the main output of these approaches.

Retinal vessel diameter is consistently expressed as arteriole-to-venule ratio (AVR). Probably, the most largely employed method is the one by Hubbard et al. [10], which computes the diameter on the basis of measurements carried out at a single, arbitrarily selected point of the vessel. Similar to more recent approaches [12–15], our method exploits vessel extraction and tracking techniques to compute diameter along an extended retinal vessel segment, rather than at a given point.

Tortuosity of the retinal vessels is also a relevant parameter to assess retinopathy [16]. A number of different methods have been proposed to assess retinal vessel tortuosity (for a review, see [17]). The two most largely employed methods compute tortuosity by using the integral of the vessel curvature [18] or the ratio between arc and cord length of the vessel segment [19]. Our newly developed method has the advantage of taking into account both the area under the curve delineated by the vessel and the directional changes along the vessel path, with improved sensitivity.

Mean-D is the main output of fractal analysis, which constitutes an operator-independent, quantitative means to assess the complexity or density of the retinal vessel branching [20]. Changes in mean-D of the retinal vascular tree are associated with hypertension [21], diabetes [22], and cerebrovascular diseases, such as lacunar stroke [23]. We recently reported clear-cut changes of mean-D values in subjects with CADASIL [24], an inherited disorder affecting the cerebral small vessels and leading to stroke and dementia.

Here, we report on the development of a semiautomated, computer-based method to assess retinal vessel morphology. The method (a) allows simultaneous and operator-independent assessment of the three parameters (AVR, tortuosity, and mean-D), (b) improves reliability of AVR by performing the assessment along extended vessel segments instead of at a single, arbitrarily selected point, (c) increases the sensitivity of tortuosity measurements, as they are carried out by a new approach, and (d) allows fully automated fractal analysis. The method implements two custom plugins (*Cioran* and* BRetina*), which are embedded into the widely used* Image-J* free software (http://rsb.info.nih.gov/ij/).

In order to test the method, we implemented it in two different clinical conditions, previously shown to present changes in AVR, tortuosity, and mean-D: hypertensive retinopathy [18, 21, 25] and CADASIL [24, 26].

## 2. Methods

### 2.1. Subjects

Four groups were considered: subjects with hypertensive retinopathy (*n* = 16), subjects with CADASIL (*n* = 11), and the respective age- and gender-matched controls (*n* = 16 and 11). Subjects with hypertensive retinopathy were consecutive patients referred to our neurology unit because of presumed cerebrovascular disease or to the ophthalmology outpatient unit of our hospital because of visual symptoms. Diagnosis of hypertensive retinopathy was carried out by an independent ophthalmologist, and the severity was scored according to the established, three-grade classification proposed by Wong and Mitchell [27]. Subjects with genetically defined CADASIL had been examined for retinopathy in a previous study [24]. The very same subjects were included in this study for the purpose of quantifying the retinal vessel changes by using the newly developed automated analysis. Control subjects were recruited among the medical or nursing staff, as well as patient relatives. From an original cohort of 54 control subjects, individuals were randomly sorted and then enrolled or rejected on the basis of matching (age ± 3 years and gender) with each of the hypertensive retinopathy or CADASIL subjects. Clinical history, including cerebrovascular risk factors, was collected to account for potential confounders (Table 1).

Retinal photographs from patients with CADASIL were obtained following approval by the local ethics committee. Fundoscopy in patients with hypertensive retinopathy was performed according to good clinical practice routine. Informed consent was obtained from all the participants. Given the noninvasiveness of the procedure, a verbal consent was deemed satisfactory.

### 2.2. Image Processing and Analysis

Fundoscopy was carried out using a digital fundus camera (Canon CR-DGI equipped with Canon EOS-40D digital camera). After pupil dilation (topical solution of tropicamide 1%), a 45° retinal photograph of one eye, centered on the region of the optic disc, was acquired. All the images were processed using* Image-J* (http://rsb.info.nih.gov/ij/) and* Frac Lac* (http://rsbweb.nih.gov/ij/plugins/fraclac/), both available as free software. The analysis was completed by means of the two custom plugins that we named* Cioran* and* BRetina*, to be embedded in* Image-J. Cioran* and* BRetina* were developed for the purpose and are herewith presented.

**Figure 1:**
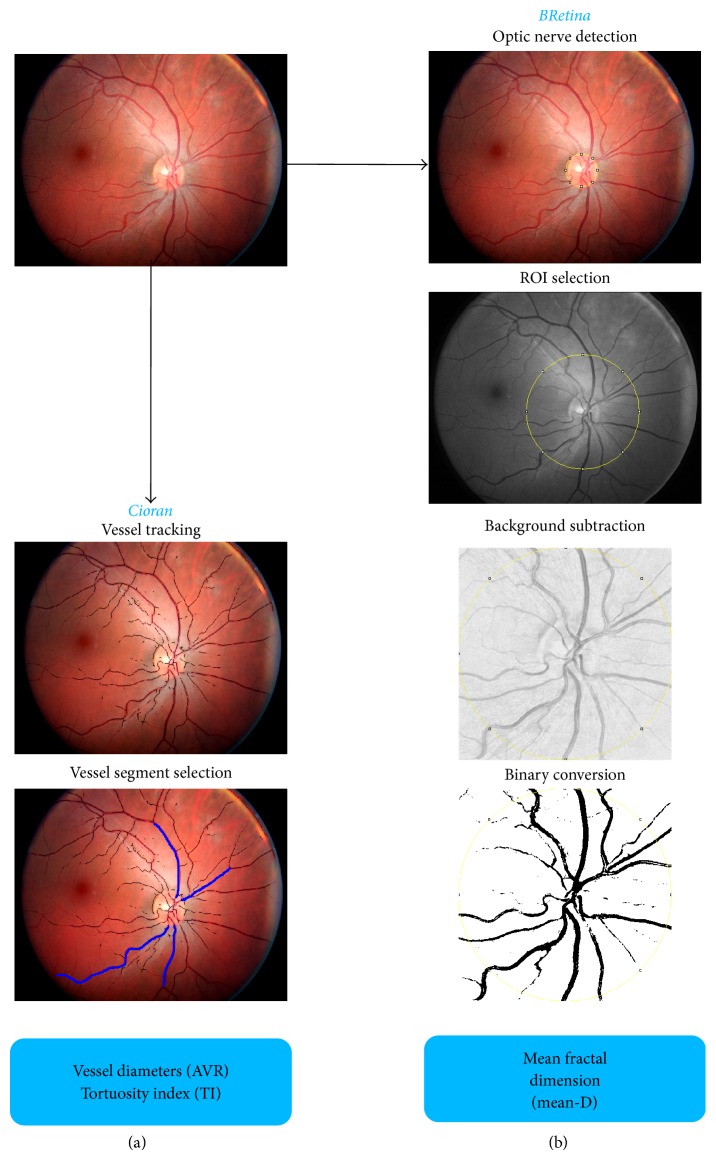
Main steps of the image processing. From an individual retinal photograph, vessel tracking and vessel segment selection is carried out by means of the* Cioran* plugin (a). The* BRetina* plugin allows the automated image processing needed to provide the mean-D value of the fractal analysis (the fundamental steps are represented in (b)). Details are in the* Box*.

*Cioran* was developed to trace the path of the main retinal vessels and to measure their thickness (AVR) and tortuosity (tortuosity index: TI) along a segment. Once launched,* Cioran* automatically identifies and tracks the visible retinal vessels (Figure 1). Among the highlighted vessels, the operator selects 2 major arterioles and 2 venules and, for each vessel, defines a segment comprised between the edge of the optic nerve and the first vascular bifurcation (Figure 1). Following this initial, arbitrary vessel and segment selection, the software computes all the parameters in an operator-independent way.

The software computes the average width of the 2 arterioles and 2 venules along the entire segments. AVR is expressed as the ratio between the average width of the 2 arterioles and the 2 venules.

On the very same vessel segments,* Cioran* computes also TI. Two parameters define TI: (a) the counts of the directional changes along the vessel path; (b) the area under the curve delineated by the same vessel segment. Both parameters are normalized by the length of the vessel segment. The TI measurements obtained by means of our new implemented software were compared with data obtained using two different methods, which define TI as the area under the curve delineated by the vessel normalized by the length of the vessel segment analyzed [18] or the ratio between arc and cord length of the vessel segment [19]. For statistical analysis, we used the average TI from the 2 arterioles and the 2 venules.

*BRetina* was developed to automatize the processing of the retinal images required by the* Frac Lac* software to perform the fractal analysis (measurement of mean-D) (Figure 1;* Box*).

*Box*

*(a) Cioran*. The workflow of* Cioran* image processing may be summarized in the following 3 steps (Figure 1): (a) vessel extraction, (b) tracking, and (c) measurement. Vessel extraction is based on iterative runs of the* Subtract Background* task [35], followed by further filtering obtained by the* Skeletonize* command, enclosed in* Image-J*. The resulting image constitutes the basis for the vessel tracking step. The operator selects the start and end points of each vessel segment to be analyzed. The *A* ^∗^ “walking” algorithm [36] is then exploited in order to obtain the basic parameters of the vessel geometry. The third step is devoted to the measurement of the diameter of the selected vessel segments. For that purpose the shortest distance between the edges of the vessel is computed for each pixel of the segment length. The average value is then taken for final result. The computation of tortuosity index is based on a combination of Bézier and Spline interpolations [37, 38], so as to obtain a regular analytical function *y* = *f*(*x*), describing the vessel path. The start and end points selected by the operator were set to *y* = 0. TI was computed taking into account (1) the area of the curve delineated by the vessel segment; (2) the number of directional changes along the vessel path, normalized by the total length of the selected vessel segments. The directional changes were defined as points where the first derivative of the analytical function is equal to zero, corresponding to the points where vessel path changes its slope. Confounding conditions that may impair the analysis in subjects with hypertensive retinopathy, such as microaneurysms, exudates, and hemorrhages, were removed by using the* Remove Outliers* filter enclosed in* Image-J* and by subtracting the resulting mask from the retinal image.

*(b) BRetina*. The* BRetina* algorithm is based on the following 4 fundamental steps, displayed in Figure 1: (a) identification of the optic nerve; (b) selection of the region of interest (ROI); (c) execution of the blood vessel filters; (d) fractal analysis. The first step includes the application of the* Variance* filter to red-free images, which allows the main retinal structures (optic nerve and vessels) to emerge from the background. The resulting image undergoes a quality check based on the* Otsu Thresholding *[39]. The software identifies the optical nerve in the most circular pattern of the image. The radius of the optical nerve is then adopted to select a circular ROI of 3.5x optic disc diameter, concentric with the optic nerve. In order to extract the retinal vessels from the background (as evaluated by* Otsu Thresholding*), the following filters (enclosed in* Image-J*) are then applied:* Subtract Background*,* Binary Conversion*,* Despeckle*, and* Outline*. The last step consists in running the external plugin* Frac Lac*, which performs the fractal analysis of the retinal vascular tree using a box counting algorithm. The whole procedure is automated and works as a single click. Nevertheless, the operator is allowed to interfere with the outcome of the automated processing at each step. For example, if the algorithm of optic nerve identification fails (e.g., due to papilledema), the operator can select and position the region of interest manually and then start over the* BRetina* workflow from that particular step.

The analyses were carried out by a trained physician (MC), blind to demographic and clinical data. A second user (CS) carried out the measurements on the entire data set to assess interrater reliability. Screenshots depicting the main steps of image processing and analysis by* Cioran* and* BRetina* are shown in Figure 1. The plugins' workflow is described in the* Box*.

### 2.3. Statistical Analysis

Wilcoxon signed rank test was used for analyzing group differences in AVR, TI, and mean-D. Sensitivity, specificity, and positive and negative predictive values of each retinal index, separately and cumulatively for the three indices, were obtained using 2 × 2 contingency tables. *K*-means was performed to classify subjects with normal versus abnormal retinal features. Intraclass correlation was used to assess interrater reliability between the two operators. Statistical analyses were performed using Graph Pad (http://www.graphpad.com/) (Wilcoxon, intraclass correlation and contingency tables) and KNIME (http://www.knime.org/) (cluster analysis).

## 3. Results

Demographics and cerebrovascular risk factors of the study subjects are reported in Table 1. Among the 16 subjects with hypertensive retinopathy, 11 had no other major disease, 3 had suffered from ischemic stroke, 1 had suffered from transient ischemic attack, and 1 had suffered from subdural hematoma. The retinopathy was graded as follows: moderate in 12/16 cases, mild in 2, and malignant in 2 of the subjects. Of the 11 subjects with CADASIL, 4 were asymptomatic, 3 exhibited a history of transient ischemic attack or stroke, 3 had chronic migraine, and 1 had seizures.

**Figure 2:**
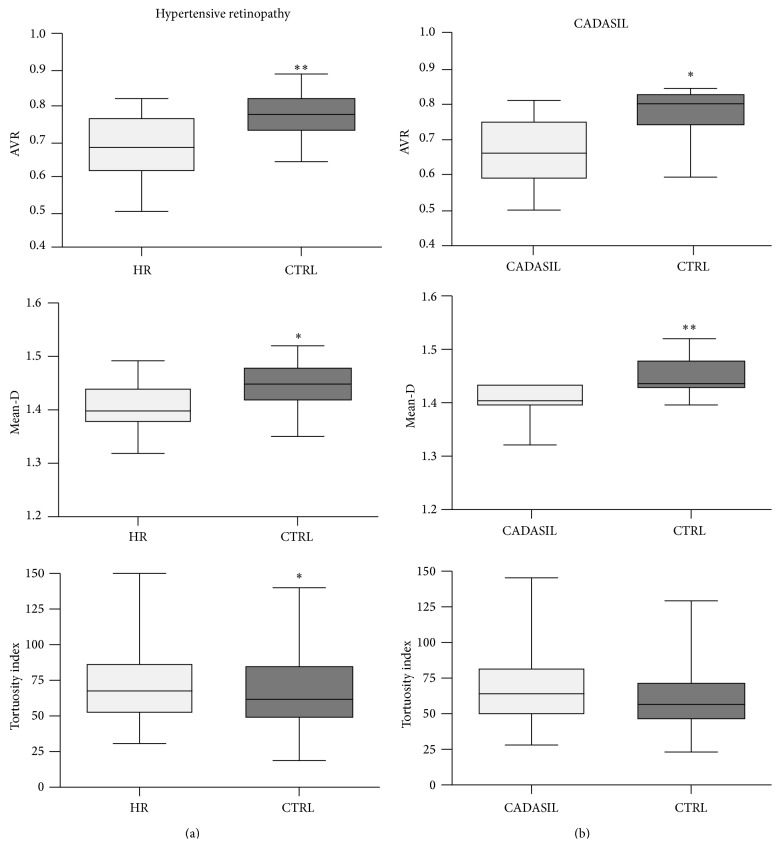
Box plots illustrating group differences in arteriole-to-venule ratio (AVR), mean fractal dimension (mean-D), and tortuosity index (TI) of the retinal vessels. Both AVR and mean-D of the subjects with hypertensive retinopathy (HR) (a) or CADASIL (b) were lower than age- and gender-matched controls. The increment of TI was significant in HR and uncertain in CADASIL group. Statistically significant differences between the groups are indicated as ∗ for *P* values ≤ 0.05 and as ∗∗ for *P* values ≤ 0.01. The boxes include data between 25th and 75th percentiles. Horizontal line in the box represents the median. The whiskers indicate the minimum and maximum values.

**Figure 3:**
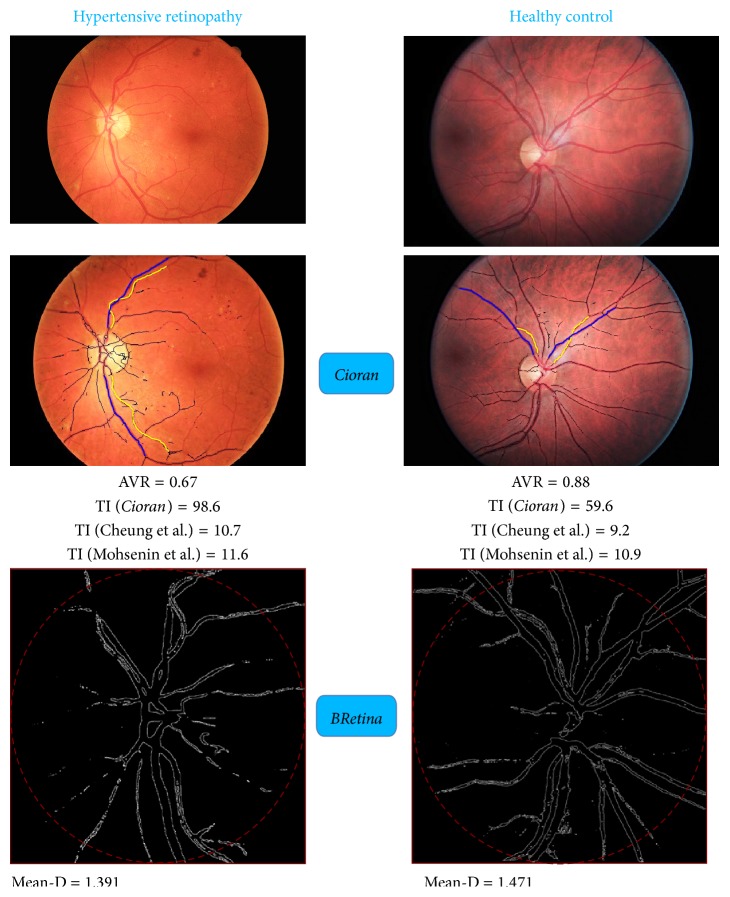
Examples of retinal image analysis of a subject with hypertensive retinopathy and matched healthy control. Arteriole-to-venule ratio (AVR), tortuosity index (TI), and mean fractal dimension (mean-D) carried out by* Cioran* and* BRetina* are reported. Tortuosity index (TI) values obtained using* Cioran* are compared to the values obtained using two different approaches to show that TI as measured using our approach is more sensitive to detect abnormal twisting of the retinal vessels. TI as measured by Cioran showed 40% relative increase in the subject with hypertensive retinopathy compared to the control, while TI as measured using the integral of the vessel curvature [18] or the ratio between arc and cord length of the vessel segment [19] showed 14% and 6% relative increase, respectively.

Analysis of the retinal images showed a marked difference between disease and control groups for all the parameters measured. AVR and mean-D were lower than control in both patients with hypertensive retinopathy and CADASIL (*P* < 0.05). TI values were higher than control in the hypertensive retinopathy (*P* < 0.05) and CADASIL (*P* = 0.08) groups. The results are reported in Table 2 and Figure 2. The following cutoffs, derived from *K*-means clustering, were applied to define the retinal indices as abnormal: <0.70 for AVR, >72 for TI, and <1.42 for mean-D. Sensitivity, specificity, and positive and negative predictive values of each retinal index separately, and of all the three indices together, are shown in Table 3. Representative fundoscopy images for each group and the main output of the analysis carried out by* Cioran* and* BRetina* are shown in Figure 3.

The interrater reliability showed an intraclass correlation coefficient of 85% for AVR, 89% for TI, and 92% for mean-D.

## 4. Discussion

We developed two custom-made plugins and embedded them into the free software* Image-J* in order to provide a novel method for quantitative, semiautomated analysis of retinal vessel features in fundoscopy images. We sought to validate the method by implementing it in two conditions known for being associated with retinal vessel changes, hypertensive retinopathy, and CADASIL [21, 24–26].

The results of the study show that the method allows us to reveal the expected retinal vessel abnormalities and to provide a quantitative assessment of the changes. Namely, we found abnormal AVR, TI, and mean-D in the hypertensive retinopathy and CADASIL groups, as compared to the matched control subjects (although in the CADASIL group the TI difference only approached statistical significance).

Our findings are in line with previous studies. Leung et al. [25] showed an inverse linear relationship between retinal vessel diameter and blood pressure in subjects with hypertension, while Ikram et al. [28] found that arteriolar narrowing was predictive of hypertension in a prospective population-based study. It is worth stressing that the AVR measurements carried out in the present study have the confidence of an approach that averages measures gathered along an extended retinal vessel segment. Most of the AVR values reported in the literature represent ratios of diameters measured at a given point. A limited number of studies measured tortuosity of retinal vessels in subjects with hypertension [29, 30]. Most of those studies are of a subjective and qualitative nature, with a few exceptions [18]. Our findings support and reinforce the findings in the literature, as the measurements performed in this study are based on a near operator-independent approach. Furthermore, our approach increases the sensitivity of the TI measurement by taking into account the number of directional changes along the selected vessel path, in addition to the area of the curve delineated by the same vessel segment. Our approach showed significant differences in TI measurements between subjects with hypertensive retinopathy and controls. Such differences were not detectable by implementing, in the very same retinal images, methods based solely on the integral of the vessel curvature [18] or the ratio between arc and cord length of the vessel segment [19] (Table 2; Figure 3). There are also very few studies of fractal analysis of the retinal vessels in subjects with hypertension. The available data obtained in adult subjects [21] or children [31] are consistent with our findings, which show a reduced complexity of the retinal vessel branching.

Reports concerning retinal vessel abnormalities in subjects with CADASIL are scant. Narrowing, sheathing, and nicking of the retinal arterioles were reported in subjects with CADASIL by means of qualitative evaluations of fundoscopy [32] or fluorescein angiography images [33]. Similar to our findings, one quantitative assessment reported reduced AVR in CADASIL [26]. In regard to TI values, to our knowledge, this is the first measurement of tortuosity carried out in CADASIL patients. Previous reports concern evaluations based on subjective scoring. Roine et al. [26] observed “straightening” of retinal arterioles in 6 and “curliness” in 1 over 38 subjects. Cumurciuc et al. [32] reported “tortuous arterioles” in 1 over 18 patients. Our findings suggest an increased tortuosity in the retinal vessel of subjects with CADASIL (Table 2). Data regarding fractal analysis of retinal vessels in CADASIL are limited to our own previous study, in which we reported clear changes of mean-D values in subjects with disease compared to matched controls [24]. The present study confirms the previous results and adds the novelty of an automatized, operator-independent method. It is worth stressing that 4 out of the 11 subjects with CADASIL were asymptomatic. Therefore, the vessel changes appear to reflect early changes.

In an exploratory fashion, we sought to verify whether the retinal indices would cluster the subjects according to the diagnosis (i.e., hypertensive retinopathy or CADASIL versus controls). AVR showed the best profile in terms of sensitivity, specificity, and positive and negative predicting values. Both TI and mean-D, in fact, showed lower predictive power compared to AVR, and the inclusion of these variables in the clustering workflow did not contribute to correctly classifying the subjects (Table 3).

The methodology reported in this study has the advantage of minimizing the analysis time and the strength of being almost operator-independent. The operator's intervention is in fact limited to the application of standardized rules, which guide in the selection of the vessel segments to be analyzed. The limited subjectivity of the entire procedure based on the* Cioran* and* BRetina* plugins is confirmed by the high interrater concordance. Therefore, the method described here is a simple and highly reproducible approach for discriminating pathological conditions characterized by changes of retinal vessel parameters.

To our knowledge, there is similar software, reported and validated in clinical setting. The software, known as Singapore I Vessel Assessment (SIVA), allows the measurement of a number of parameters from digital retinal images, including retinal vascular caliber and tortuosity [12]. Studies carried out by employing the SIVA software show changes of the measured parameters in subjects with hypertensive or diabetic retinopathy [18, 34]. Similar to SIVA, our approach allows the computation of AVR along an extended retinal vessel segment, rather than at a given point. Our method also combines simultaneous assessment of retinal vessel diameter and tortuosity with fractal analysis of retinal vascular trees. Furthermore, TI as measured by the* Cioran* software reflects both the integral of the curvature and the number of directional changes of the vessel path. The number of directional changes seems to be the relevant parameter, as the value of the integral itself (area under the curve described by the vessel path) did not give significant results in this study (Figure 3).

The method herein described may improve confidence in fundoscopy as a tool to be exploited in clinical settings or trials.

## Figures and Tables

**Table 1:** Characteristics of the study subjects.

	Age	Gender (F)	Smoking	Dyslipidemia	Hypertension	Diabetes
Hypertensive retinopathy	63 ± 15	7/16	2/16	3/16	16/16	3/16
Controls	62 ± 14	7/16	5/16	None	None	None
*P* ^∗^ (*n* = 16)	*0.85 *	*0.99 *	*0.39 *	*0.23 *	*0.0001 *	*0.23 *
CADASIL	45 ± 8	7/11	2/11	2/11	1/11	1/11
Controls	44 ± 8	7/11	3/11	1/11	None	None
*P* ^∗^ (*n* = 11)	*0.77 *	*0.99 *	*0.99 *	*0.99 *	*0.99 *	*0.99 *

Age is expressed as mean ± SD. All the other variables are expressed as ratio *n*/total.

Dyslipidemia was defined as total cholesterol ≥200 mg/dL or LDL ≥100 mg/dL or statin treatment. Hypertension was defined as elevated blood pressure (ambulatory systolic blood pressure ≥140 mmHg and/or diastolic blood pressure ≥90 mmHg) or current antihypertensive drug therapy. Diabetes was defined as fasting glucose levels ≥126 mg/dL on two different test occasions or current use of hypoglycemic agents.

^∗^Wilcoxon rank sum test (age) or Chi-squared test (all the other variables).

**Table 2:** Arteriole-to-venule ratio (AVR), mean fractal dimension (mean-D), and tortuosity index (TI) values of the study groups.

	AVR	Mean-D	TI^1^	TI^2^
Hypertensive retinopathy	0.68 ± 0.09	1.41 ± 0.04	72 ± 26	1.52 ± 1.36
Controls	0.77 ± 0.07	1.45 ± 0.04	64 ± 24	1.44 ± 0.74
*P* ^∗^ (*n* = 16)	*0.01 *	*0.04 *	*0.03 *	*0.84 *
CADASIL	0.67 ± 0.10	1.40 ± 0.04	67 ± 23	1.29 ± 1.06
Controls	0.78 ± 0.07	1.45 ± 0.04	61 ± 22	1.32 ± 0.94
*P* ^∗^ (*n* = 11)	*0.03 *	*0.002 *	*0.08 *	*0.70 *

Values are expressed as mean ± SD.

^∗^Wilcoxon signed rank test.

TI^1^: tortuosity index measured by using the method implemented in *Cioran.*

TI^2^: tortuosity index measured using the method by Cheung et al. [18].

**Table 3:** Sensitivity, specificity, and positive and negative predictive values of the retinal indices to classify subjects with hypertensive retinopathy or CADASIL.

Indices	Sensitivity		Specificity		PPV		NPV	
	HR versus controls	CADASIL versus controls	HR versus controls	CADASIL versus controls	HR versus controls	CADASIL versus controls	HR versus controls	CADASIL versus controls
AVR	68.8 (41.4–88.9)	54.5 (23.5–83.1)	87.5 (61.6–98.1)	90.9 (58.7–98.5)	84.6 (54.5–97.6)	85.7 (42.2–97.6)	73.7 (48.8–90.8)	66.7 (38.4–88.1)
Mean-D	62.5 (35.5–84.7)	99.9 (71.3–99.9)	68.8 (41.4–88.9)	36.4 (11.1–69.1)	66.7 (38.4–88.1)	61.1 (35.8–82.6)	64.7 (38.4–85.7)	99.9 (40.2–99.9)
TI	43.8 (19.8–70.1)	54.6 (23.5–83.1)	75.0 (47.6–92.6)	63.6 (30.1–88.9)	63.6 (30.1–88.9)	60.0 (26.4–87.6)	57.1 (34.0–78.1)	58.3 (27.7–84.7)
AVR + mean-D + TI	68.8 (41.4–88.9)	54.5 (23.5–83.1)	87.5 (61.6–98.1)	90.9 (58.7–98.5)	84.6 (54.5–97.6)	85.7 (42.2–97.6)	73.7 (48.8–90.8)	66.7 (38.4–88.1)

Results are expressed as % (95% CI).

AVR: arteriole-to-venule ratio; HR: hypertensive retinopathy; mean-D: mean fractal dimension; NPV: negative predictive value; PPV: positive predictive value; TI: tortuosity index.

## References

[1] Grosso A., Cheung N., Veglio F., Wong T. Y. (2011). Similarities and differences in early retinal phenotypes in hypertension and diabetes. *Journal of Hypertension*.

[2] Patton N., Aslam T., MacGillivray T., Pattie A., Deary I. J., Dhillon B. (2005). Retinal vascular image analysis as a potential screening tool for cerebrovascular disease: a rationale based on homology between cerebral and retinal microvasculatures. *Journal of Anatomy*.

[3] Wong T. Y., Klein R., Couper D. J. (2001). Retinal microvascular abnormalities and incident stroke: the Atherosclerosis Risk in Communities Study. *The Lancet*.

[4] Mitchell P., Wang J. J., Wong T. Y., Smith W., Klein R., Leeder S. R. (2005). Retinal microvascular signs and risk of stroke and stroke mortality. *Neurology*.

[5] Wong T. Y., Klein R., Sharrett A. R. (2002). Cerebral white matter lesions, retinopathy, and incident clinical stroke. *The Journal of the American Medical Association*.

[6] Liao D., Wong T. Y., Klein R., Jones D., Hubbard L., Sharrett A. R. (2004). Relationship between carotid artery stiffness and retinal arteriolar narrowing in healthy middle-aged persons. *Stroke*.

[7] de Silva D. A., Manzano J. J. F., Woon F.-P. (2011). Associations of retinal microvascular signs and intracranial large artery disease. *Stroke*.

[8] Lee A., Rudkin A., Agzarian M., Patel S., Lake S., Chen C. (2009). Retinal vascular abnormalities in patients with cerebral amyloid angiopathy. *Cerebrovascular Diseases*.

[9] Rufa A., Pretegiani E., Frezzotti P. (2011). Retinal nerve fiber layer thinning in CADASIL: an optical coherence tomography and MRI study. *Cerebrovascular Diseases*.

[10] Hubbard L. D., Brothers R. J., King W. N. (1999). Methods for evaluation of retinal microvascular abnormalities associated with hypertension/sclerosis in the Atherosclerosis Risk in Communities Study. *Ophthalmology*.

[11] Patton N., Aslam T. M., MacGillivray T. (2006). Retinal image analysis: concepts, applications and potential. *Progress in Retinal and Eye Research*.

[12] Wong T. Y., Knudtson M. D., Klein R., Klein B. E. K., Meuer S. M., Hubbard L. D. (2004). Computer-assisted measurement of retinal vessel diameters in the Beaver Dam Eye Study: methodology, correlation between eyes, and effect of refractive errors. *Ophthalmology*.

[13] Perez-Rovira A., MacGillivray T., Trucco E. VAMPIRE: vessel assessment and measurement platform for images of the REtina.

[14] Yin Y., Adel M., Bourennane S. (2013). Automatic segmentation and measurement of vasculature in retinal fundus images using probabilistic formulation. *Computational and Mathematical Methods in Medicine*.

[15] Schuster A. K.-G., Fischer J. E., Vossmerbaeumer U. (2014). Semi-automated retinal vessel analysis in nonmydriatic fundus photography. *Acta Ophthalmologica*.

[16] Keith N. M., Wagener H. P., Barker N. W. (1974). Some different types of essential hypertension: their course and prognosis. *The American Journal of the Medical Sciences*.

[17] Kalitzeos A. A., Lip G. Y. H., Heitmar R. (2013). Retinal vessel tortuosity measures and their applications. *Experimental Eye Research*.

[18] Cheung C. Y.-L., Zheng Y., Hsu W. (2011). Retinal vascular tortuosity, blood pressure, and cardiovascular risk factors. *Ophthalmology*.

[19] Mohsenin A., Mohsenin V., Adelman R. A. (2013). Retinal vascular tortuosity in obstructive sleep apnea. *Clinical Ophthalmology*.

[20] Masters B. R. (2004). Fractal analysis of the vascular tree in the human retina. *Annual Review of Biomedical Engineering*.

[21] Liew G., Wang J. J., Cheung N. (2008). The retinal vasculature as a fractal: methodology, reliability, and relationship to blood pressure. *Ophthalmology*.

[22] Cheung N., Donaghue K. C., Liew G. (2009). Quantitative assessment of early diabetic retinopathy using fractal analysis. *Diabetes Care*.

[23] Cheung N., Liew G., Lindley R. I. (2010). Retinal fractals and acute lacunar stroke. *Annals of Neurology*.

[24] Cavallari M., Falco T., Frontali M., Romano S., Bagnato F., Orzi F. (2011). Fractal analysis reveals reduced complexity of retinal vessels in CADASIL. *PLoS ONE*.

[25] Leung H., Wang J. J., Rochtchina E. (2003). Relationships between age, blood pressure, and retinal vessel diameters in an older population. *Investigative Ophthalmology & Visual Science*.

[26] Roine S., Harju M., Kivelä T. T. (2006). Ophthalmologic findings in cerebral autosomal dominant arteriopathy with subcortical infarcts and leukoencephalopathy: a cross-sectional study. *Ophthalmology*.

[27] Wong T. Y., Mitchell P. (2004). Hypertensive retinopathy. *The New England Journal of Medicine*.

[28] Ikram M. K., Witteman J. C. M., Vingerling J. R., Breteler M. M. B., Hofman A., de Jong P. T. V. M. (2006). Retinal vessel diameters and risk of hypertension: the Rotterdam Study. *Hypertension*.

[29] Daniels S. R., Lipman M. J., Burke M. J., Loggie J. M. H. (1993). Determinants of retinal vascular abnormalities in children and adolescents with essential hypertension. *Journal of Human Hypertension*.

[30] Wolffsohn J. S., Napper G. A., Ho S.-M., Jaworski A., Pollard T. L. (2001). Improving the description of the retinal vasculature and patient history taking for monitoring systemic hypertension. *Ophthalmic and Physiological Optics*.

[31] Kurniawan E. D., Cheung N., Cheung C. Y., Tay W. T., Saw S. M., Wong T. Y. (2012). Elevated blood pressure is associated with rarefaction of the retinal vasculature in children. *Investigative Ophthalmology & Visual Science*.

[32] Cumurciuc R., Massin P., Pâques M. (2004). Retinal abnormalities in CADASIL: a retrospective study of 18 patients. *Journal of Neurology, Neurosurgery and Psychiatry*.

[33] Haritoglou C., Rudolph G., Hoops J. P., Opherk C., Kampik A., Dichgans M. (2004). Retinal vascular abnormalities in CADASIL. *Neurology*.

[34] Islam F. M. A., Nguyen T. T., Wang J. J. (2009). Quantitative retinal vascular calibre changes in diabetes and retinopathy: the Singapore Malay eye study. *Eye*.

[35] Sternberg S. R. (1983). Biomedical image processing. *Computer*.

[36] Hart P., Nilsson N., Raphael B. (1968). A formal basis for the heuristic determination of minimum cost paths. *IEEE Transactions on Systems Science and Cybernetics*.

[37] de Casteljau P. (1959). *Courbes et Surfaces à Pôles*.

[38] Schoenberg I. J. (1946). Contributions to the problem of approximation of equidistant data by analytic functions. Part A. On the problem of smoothing or graduation. A First class of analytic approximation formulae. *Quarterly of Applied Mathematics*.

[39] Otsu N. (1979). A threshold selection method from gray-level histograms. *IEEE Transactions on Systems, Man and Cybernetics*.

